# De Novo Designed Peptide and Protein Hairpins Self‐Assemble into Sheets and Nanoparticles

**DOI:** 10.1002/smll.202100472

**Published:** 2021-02-15

**Authors:** Johanna M. Galloway, Harriet E. V. Bray, Deborah K. Shoemark, Lorna R. Hodgson, Jennifer Coombs, Judith M. Mantell, Ruth S. Rose, James F. Ross, Caroline Morris, Robert L. Harniman, Christopher W. Wood, Christopher Arthur, Paul Verkade, Derek N. Woolfson

**Affiliations:** ^1^ School of Chemistry University of Bristol Cantock's Close Bristol BS8 1TS UK; ^2^ School of Chemistry University of Leeds Leeds LS2 9JT UK; ^3^ School of Biochemistry University of Bristol Medical Sciences Building University Walk Bristol BS8 1TD UK; ^4^ BrisSynBio/Bristol Biodesign Institute University of Bristol Life Sciences Building, Tyndall Avenue Bristol BS8 1TQ UK; ^5^ Bristol Centre for Functional Nanomaterials School of Physics University of Bristol HH Wills Physics Laboratory Tyndall Avenue Bristol BS8 1TL UK; ^6^ School of Biological and Chemical Sciences Fogg Building Queen Mary University of London Mile End Road London E1 4QD UK; ^7^ School of Chemistry University of Glasgow 0/1 125 Novar Drive Glasgow G12 9TA UK; ^8^ School of Biological Sciences Roger Land Building, King's Buildings Edinburgh EH9 3JQ UK

**Keywords:** coiled coil, computational modeling, peptide design, protein design, self‐assembly

## Abstract

The design and assembly of peptide‐based materials has advanced considerably, leading to a variety of fibrous, sheet, and nanoparticle structures. A remaining challenge is to account for and control different possible supramolecular outcomes accessible to the same or similar peptide building blocks. Here a de novo peptide system is presented that forms nanoparticles or sheets depending on the strategic placement of a “disulfide pin” between two elements of secondary structure that drive self‐assembly. Specifically, homodimerizing and homotrimerizing de novo coiled‐coil α‐helices are joined with a flexible linker to generate a series of linear peptides. The helices are pinned back‐to‐back, constraining them as hairpins by a disulfide bond placed either proximal or distal to the linker. Computational modeling indicates, and advanced microscopy shows, that the proximally pinned hairpins self‐assemble into nanoparticles, whereas the distally pinned constructs form sheets. These peptides can be made synthetically or recombinantly to allow both chemical modifications and the introduction of whole protein cargoes as required.

## Graphical abstract

Homodimerizing and homotrimerizing α‐helical coiled‐coils are connected by a linker and joined back‐to‐back using a disulfide bond to make hairpin building blocks. Nanoparticles form when the pin is proximal to the loop, and flat sheets when pinned distal from the loop. These are made by peptide synthesis or with recombinant DNA, allowing decoration of assemblies with functional small molecules and proteins.

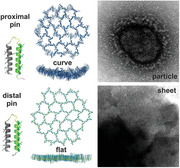

## 1 Introduction

Self‐assembling proteins form the building blocks of life to control many, if not all, cellular process. Natural self‐assembling proteins include: 1D actin filaments for cell structure and motility;^[^ ^1^ ^]^ 2D S‐layers that form protective outer barriers in some bacteria;^[^ ^2^ ^]^ 3D capsids that protect viral DNA/RNA,^[^ ^3^ ^]^ and the shells of bacterial microcompartments, which are natural nanoreactors.^[^ ^4^ ^]^ These natural scaffolds can be engineered to display other proteins to create assemblies for biotechnology.^[^ ^5^ ^]^ For example, enzyme pathways fused to viral capsid proteins self‐assemble to create nanoreactors.^[^ ^6^ ^]^ Virus‐like particles have also been engineered to display antigenic epitopes for use in vaccination,^[^ ^7^ ^]^ or adorned with targeting motifs to direct them to diseased cells.^[^ ^8^ ^]^ The toolbox of useful self‐assembling protein structures has been expanded by mutating natural protein interfaces to induce controlled self‐assembly and through de novo design.^[^ ^9^ ^]^ Such structures are usually computationally designed to form closely packed 2D arrays^[^ ^10^ ^]^, tubes,^[^ ^11^ ^]^ or 3D icosahedral particles.^[^ ^12^ ^]^ However, these beautifully ordered and near crystalline assemblies may not be amenable to decoration with large cargos, or be permeable to small molecules due to their close packed nature. Also, these engineered or designed arrays can require thermal annealing to assemble,^[10d,e]^ which may reduce or even destroy the activity of any appended proteins. Alternatively, 1D fiber assemblies can form extensive 3D gels,^[^ ^13^ ^]^ but controlling the localization of appended motifs and/or functions is limited because network formation is a stochastic process. Between these two extremes of close‐packed order and 3D entangled networks, there is space for the development of room‐temperature self‐assembling biomolecular systems that can display functional cargos and be permeable to small molecules.

Previously, we have used de novo α‐helical coiled‐coil peptides to make self‐assembled peptide cages (SAGEs).^[^ ^14^ ^]^ In SAGEs a homotrimeric coiled‐coil peptide (CC‐Tri3)^[^ ^15^ ^]^ is joined back‐to‐back with a disulfide bond to one of two halves of a heterodimeric coiled‐coil pair (CC‐DiA and CC‐DiB).^[^ ^15^ ^]^ This generates two complementary building‐blocks or hubs, A and B.^[^ ^14^ ^]^ When mixed, these self‐assemble into a hexagonal lattice that curves,^[^ ^14^ ^]^ and forms closed structures with the aid of defects.^[^ ^16^ ^]^ SAGEs have been adapted for uptake by mammalian cells as potential drug delivery vehicles,^[^ ^17^ ^]^ and decorated with immunogenic peptide sequences to make a modular vaccine platform.^[^ ^18^ ^]^ The peptide cages are permeable to small molecules, so are ideal scaffolds for nanoreactors.^[^ ^19^ ^]^ The above examples all employ synthetically derived peptides and hubs. Natural proteins, such as enzymes and whole protein antigens, are too large to incorporate into SAGEs in this way. However, the system can be adapted to make peptide‐protein fusions for recombinant production, leading to functionalized pSAGEs.^[^ ^20^ ^]^

Others have pioneered approaches to construct large coiled‐coil based nanoparticles. For example, Burkhard and co‐workers have linked a de novo homotrimeric and modified natural homopentameric coiled‐coil sequences with a short GG linker and an inter‐helix disulfide bridge proximal to the loop which self‐assemble into polyhedral nanoparticles.^[12d,g]^ The group has been particularly successful at developing these as vaccine platforms for the presentation of antigenic peptides.^[12e‐i]^ In a different concept, Marsh and co‐workers combine de novo coiled‐coil units and natural oligomeric proteins to render defined protein nanocages.^[^ ^21^ ^]^

**Figure 1:**
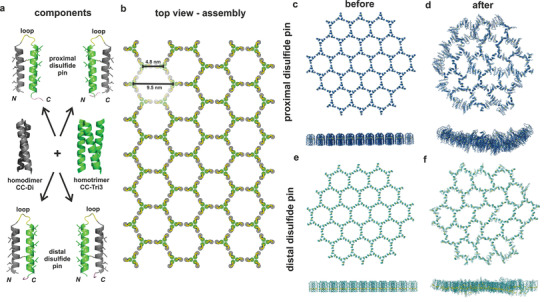
Rational design and assembly of helical hairpin peptide. a) Schematic of hairpin design. The hairpins are constructed from two 3‐heptad de novo coiled‐coil peptides based on the homodimer CC‐Di (grey, PDB code 4DZM) and the homotrimer CC‐Tri3 (green, PDB code 4DZL). These are joined by a GSGSG “loop” (yellow) and an interhelix disulfide bond “pin” (black) between the polar helical facets. This leaves the coiled‐coil forming facets exposed to engage in peptide–peptide interactions. The disulfide bond is placed either proximal or distal to the loop, which gives four possible hairpin configurations: with the dimer N terminal (left) or C terminal (right), with proximal (top) and distal (bottom) disulfide bonds. Full amino acid sequences for the designs are in Table 1. b) Cartoon of the envisaged hexagonal network formed when the hairpins self‐assemble via their coiled‐coil interfaces, labelled with intervertex dimensions measured from pre‐simulated hexagons. c–f) Snapshots from molecular dynamics (MD) simulations of all‐atom models of patches of assembled peptides. These were taken c,e) before and d,f) after 100 ns of MD under periodic boundary conditions using explicit TIP3P water, pH 7.0, 298 K, 0.15 m NaCl. Snapshots from assemblies of the c,d) proximally pinned hairpins (Movie S1, Supporting Information) and e,f) distally pinned hairpins (Movie S2, Supporting Information).

Here we present self‐assembling hairpin designs that form two distinct supramolecular assemblies; namely, closed nanoparticles and extended sheets. These designs combine ideas from the SAGEs and Burkhard's nanoparticles. Specifically, homodimeric (CC‐Di) and homotrimeric (CC‐Tri3) blocks^[^ ^15^ ^]^ are joined by a flexible linker, and pinned back‐to‐back with a disulfide bond (**Figure** **1**). Computational modeling indicates that these should fold into stable hairpins that can be arrayed hexagonally. Moreover, and distinctively, the models suggest that the position of the disulfide may influence the supramolecular assembly profoundly: a pin proximal to the loop leads to curved arrays, which could close to form particles, whereas a distal pin restricts curvature, potentially leading to extended sheets. These extremes are confirmed experimentally using a variety of advanced microscopy methods for hairpins made by peptide synthesis and when produced recombinantly. The validated designs offer de novo scaffolds with potential to display a range of functionalities for application in imaging, cell targeting, nanoreactors, drug delivery, and modular vaccines.

## 2 Results and Discussion

### 2.1 Design of α‐Helical Hairpin Peptides

Our design strategy used a GSGSG sequence to link two self‐assembling coiled‐coil 3 heptad building blocks: homodimeric (CC‐Di) and homotrimeric (CC‐Tri3).^[^ ^15^ ^]^ This gives two possible linear peptide sequences: CC‐Di–link–CC‐Tri3 and CC‐Tri3– link–CC‐Di, abbreviated to DT and TD respectively. These were pinned back‐to‐back with a disulfide bond between cysteines placed at one of two complementary *f* positions either proximal (Cys15 & Cys35) or distal (Cys8 & Cys41) to the linking loop. In combination, this gives four possible constructs illustrated in Figure 1 and their sequences listed in **Table** **1**. To assess any differences in the placement of the disulfide pins, we constructed all‐atom models for 19‐hexagon patches of assembled arrays for the two different pin positions and subjected these to molecular dynamics (MD) simulations in water (Figure 1c–f, Movies S1 and S2, Supporting Information). Intriguingly, the patch of the proximally pinned hairpins (_pep_HP–DT_prox_) curved in the 100 ns simulation. This resulted in the N and C termini of the peptides being presented on the convex face and the loops on the concave side. Projection of this curvature suggested that the arrays could close to form a nanoparticle 71 ± 11 nm in diameter, i.e., like the SAGEs (Figure S1a, Supporting Information). In contrast, simulations of arrays of distally pinned hairpins (_pep_HP–DT_dist_), the 19‐hexagon patches remained relatively flat throughout the trajectories, with no preferred direction or magnitude of curvature (Figure S1b, Supporting Information). This suggested that these peptides might form flat extended sheets. The cysteines in the model assembly were replaced with glutamine, so that the hairpin building blocks could no longer form the disulfide pin. These modeled “C to Q” hairpin constructs did not remain flat, but instead flexed out of plane with no preferred direction of curvature, indicating that the assembly was unstable in the absence of the disulfide pins (Movie S3 and Figure S1c,d, Supporting Information).

**Table 1:** Peptide sequences synthesized using solid‐phase peptide synthesis. Peptide name and corresponding amino acid sequence aligned with numbering of amino acid position (1 to 51) and coiled‐coil register (*a* to *g*). Sequences based on a basis set of coiled‐coils,^[^ ^15^ ^]^ with the sequence based on the homodimer (CC‐Di) shown in grey, and the sequence based on the homotrimer (CC‐Tri3) shown in green. Cysteine mutations introduced at f positions in order to form a disulfide bonded hairpin are underlined. Linkers (G, GSGSG) and chromophore tags (GYY) are shown in black

Name	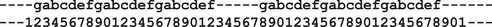
_pep_HP–DT_dist_	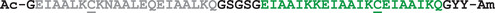
_pep_HP–DT_prox_	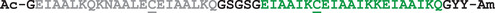
_pep_HP–TD_dist_	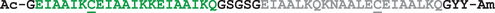
_pep_HP–TD_prox_	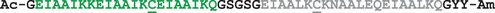

These simulations gave the first indication of differences between the proximally and distally pinned systems. We rationalize this in terms of different flexibilities in the constructs. For the proximal pin, the helical N and C termini have more freedom to explore space and move apart, resulting in wedge‐shaped building‐blocks, narrow at the loop and wider at the termini. A distal pin reduces this freedom and constrains the assembly to a flatter topology.

### 2.2 Peptide Hairpins Assemble into Particles and Sheets as Predicted

All four peptide hairpin designs (Figure 1a and Table 1) were synthesized and purified by HPLC. The constructs were oxidized with iodine to form the disulfide pin, and subject to Ellman's test^[^ ^22^ ^]^ to confirm disulfide bond formation. The peptides were confirmed as monomers by nanospray ionization mass spectrometry (Figures S2 and S3, Supporting Information, sequences and mass data in Table S1, Supporting Information). As judged by a change from clear to cloudy, all four peptides began to assemble as soon as they were hydrated in aqueous buffer. As a result, the samples scattered light and it was not possible to record reliable circular dichroism spectra to confirm α‐helical assemblies.

**Figure 2:**
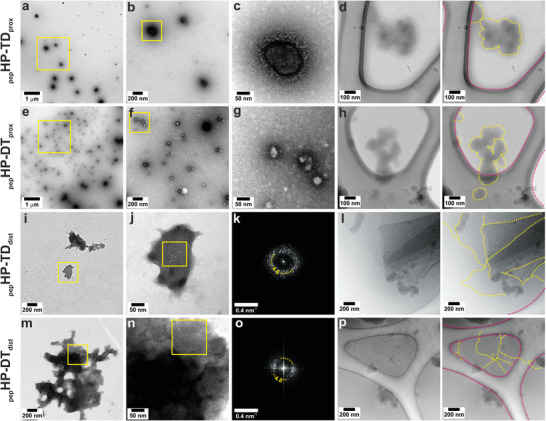
Negative stained TEM, FFTs, and cryo‐TEM of peptide hairpin assemblies. Pin proximal to the loop: a–d) _pep_HP–TD_prox_ and e– h) _pep_HP–DT_prox_, formed closed structures. Pin distal to the loop: i–l) _pep_HP–TD_dist_ and m–p) _pep_HP–DT_dist_, formed flat structures. Negative stained samples at a,e) low, b,f,i,m) intermediate, and c,g,j,n) high magnification. Fourier transforms (FFT) from distally pinned hairpins, area highlighted in j) FFT shown k) from _pep_HP–DT_dist_, and area highlighted in n) FFT shown in o) for _pep_HP–TD_dist_. d,h,l, p) Cryo‐TEM images (unannotated on the left, peptide structure edges highlighted in yellow dotted lines on the right to distinguish them from the lacey carbon support delineated in pink). Hairpin peptide aliquots (100 × 10^–6^ m) were assembled for 1 h in HBS (25 × 10^–3^ m HEPES, 25 × 10^–3^ m NaCl, pH 7.2).

Instead, we visualized the assemblies by negative‐stain transmission electron microscopy (TEM) and cryogenic transmission electron microcopy (cryo‐TEM) (**Figure** **2**). With either CC‐Di or CC‐Tri3 as the N‐terminal block, the proximally pinned peptides _pep_HP–DT_prox_ and _pep_HP–TD_prox_ respectively, formed closed nanoparticle structures (Figure 2a–h). In TEM, the particles seemed to have a nonuniform size distribution. As the particle edges were difficult to discern in both the negatively stained and cryo‐TEM images, we assessed particle size distribution by atomic force microscopy (AFM) (see below). All of the analogous peptides with the distal pin, _pep_HP–DT_dist_ and _pep_HP–TD_dist_, formed sheet‐like structures, extending for >100 nm in the *xy* dimension (Figure 2i–p). Thus, these initial experimental data support the MD simulations. Further, as there was no discernible difference in assemblies produced by the constructs with CC‐Di or CC‐Tri3 units placed first in the sequence, all further data presented had the CC‐Di sequence at the N terminus, i.e., HP‐DT constructs.

**Figure 3:**
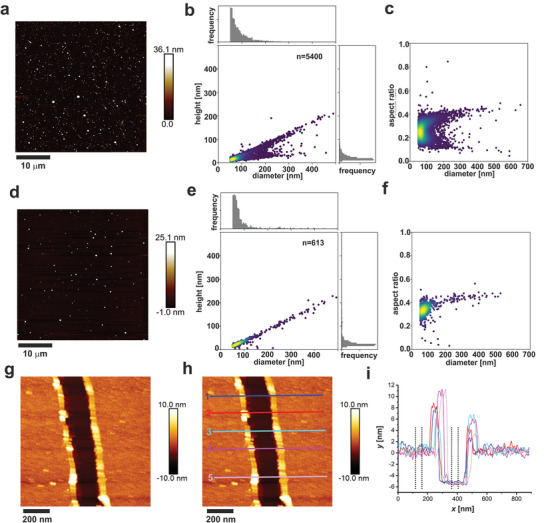
AFM grainsizing and height measurements of hairpin peptides. Proximally pinned _pep_HP–DT_prox_ (100 × 10^–6^ m) were assembled in HBS (25 × 10^–3^ m HEPES, 25 × 10^–3^ m NaCl, pH 7.2) for a–c) 1 h and d–f) 24 h, then deposited onto mica. Images were recorded using peak force atomic force microscopy (PF‐AFM, a,d). Particle height and diameter data were extracted using a particle analysis script (link available in Supporting Information) and plotted to show height versus diameter b,e) and diameter versus aspect ratio c,f) (areas colored in red were the highest populated, those in blue the lowest populated). In b,c) *n* = 5400 particles, in e,f) *n* = 613 particles. Distally pinned _pep_HP–DT_dist_ was assembled as above for 1 h, deposited on mica and imaged using tapping mode AFM (TM‐AFM, g). Height profiles were measured across a tear using Nanoscope analysis v1.5. (positions annotated on (h) and shown in (i)) where dotted lines indicate the area averaged to get the height difference between the mica substrate and the assembled peptide sheet, shown in Table S2 (Supporting Information), *n* = 5, ± standard deviation.

Next, we used AFM to probe the nature and dimensions of the assemblies in more detail, **Figure** **3**. First, at 1 h postassembly, the proximally pinned peptide _pep_HP–DT_prox_, formed nanoparticles with an average maximum height of 27 ± 22 nm and average diameter 101 ± 58 nm (*n* = 5400) Figure 3a–c. The large ranges reflected a bimodal distribution of the aspect ratios, centered on ≈0.1 and ≈0.4. After 24 h, the particles had approximately the same diameter (104 ± 79 nm), but only the thicker particles were apparent, with a height average of 39 ± 38 nm and aspect ratio 0.37, Figure 3d–f. Though the distributions were broad, these data were consistent with the particle sizes seen in TEM and cryo‐TEM, and estimated from the modeling (71 ± 11 nm), and showed that the particle diameters were stable over time. In addition, the experimental data indicated maturation of the particles between 1 and 24 h. We cannot be sure what this is due to, but posit that recruitment of peptides to the structures over time may result in organization into a multilamellar, stiffened particles, with the same diameter as seen in the earlier assembly.

**Figure 4:**
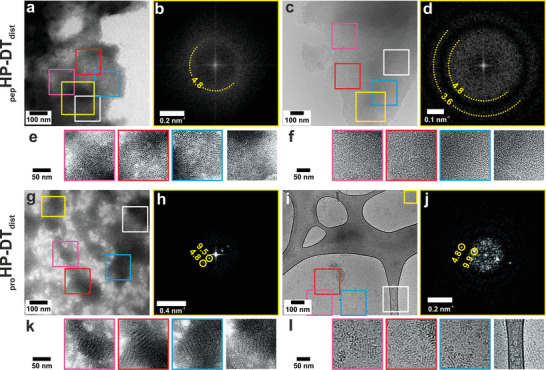
Negative stain TEM, cryo‐TEM and FFTs showing peptide and protein hairpin sheet structures. Distally pinned hairpin peptide _pep_HP–DT_dist_ a) negatively stained and b) FFT of area indicated in yellow, and c) cryo‐TEM and d) FFT of area indicated. Areas highlighted in (a) from negative stain are shown in e), and highlighted in (c) from cryo‐TEM are shown in f), (enlarged 2× and brightness and contrast enhanced). Protein version _pro_HP–HT_dist_ g) negatively stained and h) FFT of area indicated, and i) cryo‐TEM and j) FFT of area indicated. Areas exhibiting striped features of the assembled protein hairpins highlighted in (g) from negative stain are shown in (k), and highlighted in i) from cryo‐TEM are shown in l) (enlarged 2× and brightness and contrast enhanced). Features in FFTs are labelled with their corresponding distance in real space in nm. Hairpin samples (100 × 10^–6^ m) were hydrated in HBS (25 × 10^–3^ m HEPES, 25 × 10^–3^ m NaCl, pH 7.2) for 1 h.

AFM of the distally pinned peptide, _pep_HP–DT_dist_, revealed thin (5.6  ±  0.2 nm, Table S2, Supporting Information *n* = 5 ± standard deviation) sheets (Figure 3g–i and Figure S4, Supporting Information). This is similar to the expected height of one hairpin from termini to the loop, which is ≈5 nm. This value, and the tight distribution of the experimental data support the hypothesis that these peptides form unilamellar sheets. It was not possible to discern the lattice clearly in AFM (Figure S5, Supporting Information), which could be due to tip resolution, or flexibility and thermal motions in the assembly. However, fast Fourier transform (FFT) analysis of further TEM and cryo‐TEM images (**Figure** **4**b,d) revealed regular structures with spacings of 4.8 nm. This corresponds closely to the expected inter‐vertex (inter‐trimer) distances of the hexagonal lattice, which span four helices each just over ≈1 nm in diameter (Figure 1b).

### 2.3 Mixed Assemblies Can Be Made, but Preassembled Structures Do Not Exchange Peptide Components

To test for peptide mixing during assembly and for exchange postassembly, we made two fluorescently labeled variants of _pep_HP–DT_dist_ and performed correlative light and electron microscopy (CLEM). We focused on this design as the sheets were much larger than the nanoparticles formed by the proximally pinned constructs, which made correlating the light and electron microscopy for individual sheets clearer than for the particles. The peptides had N‐terminal 5(6)‐carboxyfluorescein (green) and 5(6)‐carboxytetramethylrhodamine (red) labels, Table S2 (Supporting Information). First, the labeled peptides were mixed when unfolded in 50% aqueous acetonitrile and freeze dried. When hydrated, these samples formed mixed assemblies as judged by the coincidence of the fluorescence from the two labels and electron density in CLEM, Figure S6a–f (Supporting Information). This demonstrates that differently decorated hairpin peptides can be combined into the same assembly. Second, green‐ and red‐labeled hairpins were hydrated for 1 h separately before mixing, incubated for 1 h further, and then prepared for microscopy. In this case, the CLEM revealed distinct regions of green and red fluorescence, indicating that once assembled, the structures were stable and did not exchange peptide modules. Thus, despite their flexible construction, the hairpin peptides form robust and stable sheet assemblies from solution.

### 2.4 Hairpin Redesign for Protein Expression

Next, we sought to add functional proteins to the hairpin constructs. For this, we turned to the expression of synthetic genes in *Escherichia coli*. We designed genes for two constructs, _pro_HP–DT_prox_ and _pro_HP–DT_dist_, that harbored a 5’ multiple cloning site and a His‐tag^[^ ^23^ ^]^ at the 3’ end (Figure S7, Tables S1 and S5, Supporting Information); the prescript “pro” refers to the recombinantly expressed protein constructs. These were overexpressed in SHuffle T7 cells, which are engineered to allow disulfide bond formation in their cytoplasm, then purified and characterized by SDS‐PAGE and confirmed as monomers by nanospray ionization mass spectrometry (Figure S8a–f, Supporting Information). Ellman's test^[^ ^22^ ^]^ confirmed that the molecules contained disulfide bonds. CLEM imaging of cell sections immunolabelled for the His‐tag revealed protein dispersed within the cells rather than forming inclusion bodies (Figure S9, Supporting Information).

Whereas the proximally pinned synthetic peptide hairpins formed spherical structures (Figure 2a–h), the recombinant variant, _pro_HP–DT_prox_ formed aggregates (Figure S10a, Supporting Information pH 7.2). However, like the peptide variant (Figure 4a–f), the distally pinned protein hairpin, _pro_HP–DT_dist_ formed sheets (Figure 4g–l). Interestingly, these were smaller and had a clear ultrastructure, in the form of stripes. These stripes were apparent in both negative stain TEM (Figure 4e,k) and cryo‐TEM (Figure 4f,l), and thus they are not a drying artefact and must reflect some underlying structure. FFTs of these revealed spots, which correspond to lines in real space separated by ≈4.8 nm and ≈9.7 nm in both negative stain and cryo‐TEM (Figure 4h,j). As described above, this in consistent with the expected vertex‐to‐vertex spacing of ≈4.8 nm along the hexagon side, and of ≈9.5 nm across the twofold symmetry axis (Figure 1b). When compared to the peptide design, the protein has a flexible charge neutral region N terminal to the hairpin sequence, and a C‐terminal His‐tag. It is possible that the small sheets are dimerizing through the His‐tag,^[^ ^24^ ^]^ and forming slightly overlapping sheets, leading to Moiré fringes.^[^ ^25^ ^]^ Alternatively, these protein assemblies may form corrugated or stripy structures. Therefore, we investigated how the protein patches behaved in silico and in vitro under different experimental conditions.

### 2.5 pH Alters Protein Hairpin Assembly Structure

**Figure 5:**
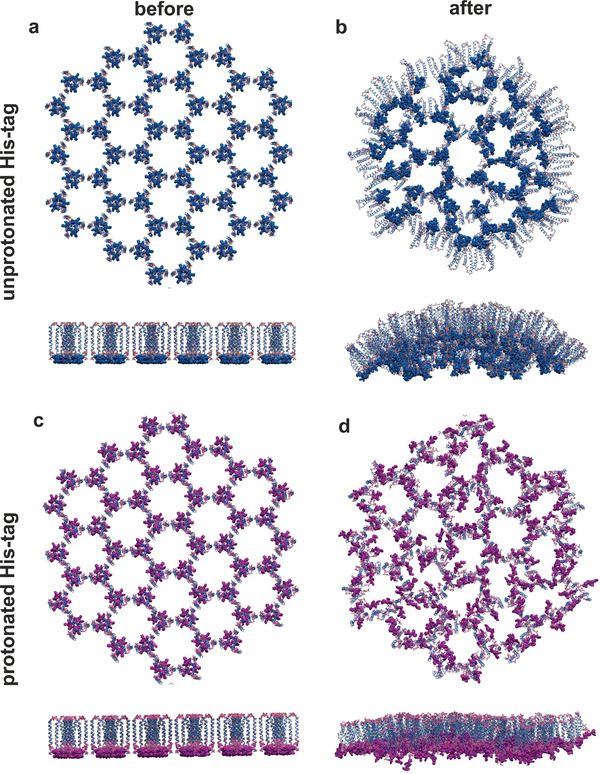
Snapshots from all atom MD simulations of 19 hexagon protein hairpin patches. Distally pinned protein hairpins (_pro_HP–DT_dist_) with a,b) unprotonated (Movie S4, Supporting Information) and c,d) protonated (Movie S5, Supporting Information) His‐tags. Snapshots taken (a,c) before (blue, Movie S4, Supporting Information) and b,d) after (magenta, Movie S5, Supporting Information) 100 nm of MD simulation under periodic boundary conditions using explicit TIP3P water, pH 7.0, 298 K, 0.15 m NaCl.

We constructed all‐atom models for 19 hexagon patches of assembled arrays for the distally pinned and His‐tagged protein _pro_HP–DT_dist_. The p*K*a of the histidine side chain is near physiological pH.^[^ ^26^ ^]^ MD simulations were run for the distally pinned protein hairpin _pro_HP–DT_dist_ with unprotonated and protonated His‐tags as described above (**Figure** **5** and Movies S4 and S5, Supporting Information). When unprotonated, the patch curved after 100 ns simulation (Figure 5b). The curvature was opposite to that seen for the proximally pinned peptide, with the loop on the convex side in unprotonated _pro_HP–DT_dist_ rather than on the concave side as was seen for _pep_HP–DT_prox_. The protonated constructs maintained a flat trajectory throughout the 100 ns simulation (Figure 5d), as was seen for the distally pinned peptide _pep_HP–DT_dist_.

The assembly behavior of the protein hairpins was tested by hydration in HBS at pH values between 6.0 and 8.0 (Figure S10, Supporting Information). The distally pinned protein formed extensive sheet like structures at low pH (<pH 6.5), and 3D aggregates at higher pH (8.0). Stripes appeared in the images of the _pro_HP–DT_prox_ structures, but at slightly lower pH (6.5) when compared to the _pro_HP–DT_dist_ (7.2). Aggregates of _pro_HP–DT_prox_ were formed at low and high pH, indicating that the proximally pinned protein was not able to form stable structures. Modeling indicated that deprotonation of the His‐tag induced the opposite sense of curvature to that caused by the proximal pin position. Thus _pro_HP–DT_dist_ may be too flexible, and the opposite sense of curvature induced by protonation versus pin position destabilized the assembly leading to an aggregation‐based failure mechanism. The striped assemblies may represent a transition between the low pH “peptide‐like” structure and the high pH “unprotonated curved” structures, probably induced by partial protonation of the His‐tag. As its assembly was more stable, only _pro_HP–DT_dist_ was investigated further.

### 2.6 Successful Self‐Assembly of Hairpins When Fused to Large Cargos

Fusions of the hairpin to the fluorescent protein sfGFP (≈28 kDa) (Figure S7 and Tables S1 and S5, Supporting Information) were expressed in SHuffle T7 cells and purified as described above (Figure S8, Supporting Information). The fluorescence in CLEM images of cell sections indicates that the sfGFP cargo folds in the cytoplasm (Figure S11, Supporting Information). Again, we were not able to see any assembled structures within the cytoplasm. If the hairpins were able to self‐assemble in cells, it may be that their open network structure is not electron dense enough to resolve in thin‐section in TEM, so these constructs were also characterized in vitro. Ellman's test^[^ ^22^ ^]^ confirmed that the molecules contained disulfide bonds after purification. Nanospray mass spectrometry and SDS‐PAGE indicated that _pro_GFP–HP–DT_dist_ was monomeric (Figure S8g–i, Supporting Information). When hydrated in HBS, we observed flat sheets formed by the cargo laden hairpins (Figure S12, Supporting Information). This demonstrates that distally pinned hairpins are amenable to being decorated with large proteins.

## 3 Conclusion and Future Outlook

In summary, we have described the design, modeling, synthesis, assembly and characterization of two types of de novo designed peptide hairpins. In these, sequences for coiled‐coil dimers and trimers are joined through a flexible linker. The hairpins are then pinned, using disulfide bridges either proximal to or distal from the linker. The aim being to expose the hydrophobic seams of the coiled‐coil segments and promote assembly of the hairpins into hexagonal arrays. The two pin positions have profoundly different effects on the topology of the supramolecular assemblies formed. The proximal pins lead to closed, spherical objects on the order of ≈100 nm in diameter, whereas the distal pin results in sheet‐like assemblies consistent with a monolayer of folded, self‐assembled hairpins. These structures are observed in a range of advanced microscopies, and the proposed mechanism of formation are supported by extended molecular dynamics simulations. Specifically, the proximal pin allows splaying of the helical termini, which in turn leads to curved arrays that can close, whereas the distal pins give a more tightly constrained hairpin structure, consistent with building blocks required to make flat sheets.

As noted above, others have developed similar self‐assembling peptide‐ and protein‐based nanoparticles^[^ ^12^ ^]^ or sheets.^[^ ^10^ ^]^ So what are the differences and advantages to our hairpin system? First, by including two points (loop and pin) that define the relative positions and rotational freedom of the two coiled‐coil components, we are able to control the topology of the self‐assembled structures by design in a single system to render closed nanoparticles or extended sheets. This level of dual control is encouraging for future biomaterials design using relatively short linear peptides. Second, although this is true for some other systems, the relatively short lengths of our hairpin designs and the minimal postsynthesis processing allows them to be made both synthetically and recombinantly. This has allowed us to decorate assemblies chemically, and through fusions to functional natural proteins; and to explore additional properties of the system. For example, experiments with fluorophore‐labeled synthetic hairpin peptides show that coassemblies can be made when starting with mixtures of unfolded peptide variants. However, once formed, there is no interchange between assembled structures. This approach could be used to add other functional moieties, e.g., for drug delivery and targeting diseased cells. Extending this to recombinantly produced hairpin–protein fusions, which express and purify well, could allow the production of nanoparticles or sheets with combinations of small molecule and protein cargoes. Third, the fabric of the system is potentially modular, and we envisage that other de novo coiled‐coil units could be swapped in to change the homomeric components used here.^[^ ^27^ ^]^ For instance, as we have demonstrated for the foregoing SAGE system,^[^ ^14^ ^]^ the homodimer sequence on the hairpins could be changed to a heterodimer pair.^[^ ^27^, ^28^ ^]^ This would afford an additional level of control on the assembly.

In all of these respects, the linear peptide hairpins that we describe provide de novo modules to add to the growing toolkit of components for the rational construction of biomaterials.^[^ ^9^ ^,12c,^ ^29^ ^]^ That said, how subtle differences in peptide design translate to relatively small changes in module structure and then to completely different assembly topologies are both alarming and encouraging. It is of concern because it highlights how careful the design process must be in order to arrive at a targeted design, and that designs must be tested when appending functionality to any self‐assembling building‐block. However, it is also exciting, as it opens considerable possibilities for designing a wider range of biomaterial structures and functions. Whatever your stance, the rational and computational de novo design of biomaterials remains both challenging and full of potential.

## 4 Experimental Section

A detailed description of the materials and methods is included in the Supporting Information.

### MD Simulation

The coiled‐coil helices were arranged in a 19 hexagon patch.^[^ ^14^ ^]^ The disulfide pins were constructed by hand and aligned, and a His‐tag added if necessary, in InsightII (Accelrys), and minor discrepancies in disulfide bond lengths fixed to 2.0 Å with an in‐house Fortran program. A box 4 nm larger than the patch assembly was filled with TIP3P water, sodium and chloride ions (0.15 m NaCl) and H^+^ consistent with pH 7.0 (≈3 million atoms). This was parameterized with the Amber‐99SB9ildn force field, before undergoing 5000 steps of energy minimization. The GROMACS‐4.6.7 suite was used to perform simulations as NPT ensembles at 298 K using periodic boundary conditions. The simulations were integrated with a leap‐frog algorithm over a 2 fs time‐step, constraining bond vibrations with the P‐LINCS method. Structures were saved every 0.1 ns for analysis and run over 100 ns. Molecular graphics manipulations and visualizations were performed using InsightII, VMD‐1.9.1 and Chimera‐1.10.2 and movies made using VMD.

### Peptide Synthesis and Purification

A low‐loading rink amide resin support was used in a Liberty microwave peptide synthesizer for solid phase peptide synthesis.^[^ ^30^ ^]^ Fmoc‐protected amino acids (0.1 × 10^–3^ m) were coupled (4.5 eq chloro‐1‐hydroxybenzotriazole (Cl‐HOBt), 5 eq. *N,N'*‐ diisopropylcarbodiimide (DIC) in 7 mL dimethylforamide (DMF) at 25 °C for 2 min, then at 25 W microwave irradiation at 50 °C for 5 min), washed (3 × 7 mL DMF), deprotected (7 mL 20% (v/v) morpholine in DMF at 20 W and 75 °C for 5 min), and washed (3 × 7 mL DMF) before adding the next Fmoc protected amino acid.

Peptides were capped using 3 eq. acetic anhydride and 4.5 eq. *N,N*‐diisopropylethylamine (DIPEA) in 10 mL DMF for 30 min at room temperature (see Supporting Information for details on fluorescent labeling). The peptides were washed (3 × 5 mL DMF, 3 × 5 mL dichloromethane (DCM)), dried, and cleaved (9.5 mL trifluoroacetic acid (TFA), 250 µL water and 250 µL triisopropylsilane (TIPS), 3 h). The resin was removed by filtration and excess TFA evaporated. The crude peptide was precipitated (30 mL diethyl ether (Et_2_O), 4 °C), pelleted by centrifugation, the Et_2_O discarded, the pellet dissolved in 50% (v/v) aqueous acetonitrile (MeCN), and freeze dried. Peptides were purified by reverse phase‐high pressure liquid chromatography (RP‐HPLC, buffer *A* = 0.1% (v/v) TFA in water, buffer *B* = 0.1% (v/v) TFA in MeCN). ≈10 mg was dissolved in 3 mL (70% buffer A,30% buffer B) and reduced using 10 eq tris(2‐carboxyethyl)phosphine (TCEP, ≈8 mg), before injection onto a Phenomenex C8 column. A gradient from 30% to 70% buffer B was applied over 30 min, and absorbance monitored at 220 and 280 nm to monitor elution of the peptide, which was checked for purity, pooled and then freeze dried. The reduced peptide was dissolved (1 mg mL^−1^) in 20% (v/v) aqueous acetic acid, and 1–2 µL iodine solution mixed in to oxidize the cysteines and form the disulfide pin. After 30–60 min, excess iodine was quenched with 100 µL aliquots of 100 × 10^‐3^ m sodium thiosulfate^[^ ^31^ ^]^ (color change from yellow to clear). This was dried and purified by RP‐HPLC as described above. Peptides were analyzed for purity using analytical RP‐HPLC and nanospray mass spectrometry (Waters SYNAPT G2S) injected using an Advion TriVersa Nanomate autoinjector. Disulfide bond formation was confirmed using a quantitative Ellman's test.^[^ ^22^ ^]^

### Peptide and Protein Assembly

Peptide aliquots (1–10 nmol) were dissolved in 4‐(2‐hydroxyethyl)‐1‐piperazineethanesulfonic acid (HEPES) buffered saline (HBS, 25 × 10^‐3^ m HEPES, 25 × 10^‐3^ m NaCl, pH 7.2) to a final peptide/protein concentration of 100 × 10^‐6^ m, and incubated for 1–24 h.

### Atomic Force Microscopy (AFM)

5 µL in vitro assembled sample was dropped onto freshly cleaved mica and washed (3 × 100 µL water) and dried. PeakForce AFM (PF‐AFM) imaging was done on Bruker Multimode with a Nanoscope V controller with Picoforce extender, and tapping mode (TM‐AFM) on a Bruker Multimode with a Quadrexed Nanoscope 3D controller.

### Transmission Electron Microscopy (TEM)

5 µL in vitro assembled sample was dropped onto a TEM grid, washed (3 × 10 µL water) and dried. For fluorescence imaging for correlative light and electron microscopy (CLEM), grids were then imaged on a Leica DMI4000B inverted epifluorescence microscope using a 63× oil objective lens with a numerical aperture 1.4. These were then negatively stained with 1% (w/v) uranyl acetate, washed (10 µL water), and dried. Samples were imaged on a Tecnai 12‐FEI 120 kV BioTwin Spirit TEM (tungsten filament, 120 keV) and a FEI Eagle 4k × 4k CCD camera. TEM images were processed and aligned for CLEM using ImageJ.^[^ ^32^ ^]^

### Cell Sample Thin Sections

2 µL of cell pellet was high pressure frozen on an EMPACT2 + RTS system, and freeze substituted with minimal stain and resin as per Lee et al.^[^ ^33^ ^]^ Cured resin was 70 nm sectioned using an EM UC6 microtome and 45° diamond knife and collected onto TEM grids. For immunolabelling, sections were blocked (1% (w/v) bovine serum albumen (BSA) in phosphate buffered saline (PBS, 140 × 10^–3^ m NaCl, 2.7 × 10^–3^ m KCl, 10.1 × 10^–3^ m Na_2_HPO_4_, 1.8 × 10^–3^ m KH_2_PO_4_ pH 7.4, 2 × 5 min). Blocked sections were labeled with a primary antibody (1:500 6x‐His‐tag monoclonal mouse/IgG2b antibody in 1% BSA in PBS, 1 h), washed (3 × 5 min in 1% BSA in PBS), incubated with a fluorescent secondary antibody (DyLight 488 labelled secondary polyclonal goat anti mouse IgG antibody, 1 µg mL^−1^ in 1% BSA in PBS, 1 h), then washed in 1% BSA in PBS followed by water before drying. Fluorescence imaging (described above) was done before TEM.

### Cryo‐TEM

5 µL in vitro assembled sample was blotted and plunge frozen onto Lacey carbon grids using a Leica EM GP automatic plunge freezer and stored in liquid N_2_. Frozen samples were loaded into a Gatan CryoTransfer specimen holder and imaged using a Tecnai 20 FEI 200 kV Twin Lens scanning transmission electron microscope (LaB_6_ filament, 200 keV) on a FEI Eagle 4k x 4k CCD camera. Higher resolution images were recorded on an FEI Talos Arctica equipped with a 200 kV X‐FEG and a Gatan GIF Quantum LS energy filter on a Ceta 16 M CCD detector or Gatan K2 DED. In addition to ImageJ, cryo‐TEM images were processed using the Gatan Microscopy Suite (GMS) 3 software.

### Protein Expression and Purification

Sequences were optimized for production in *E. coli* (Table S5, Supporting Information), ordered from GeneART, and transferred to pET3a (lactose control) and TBAD (arabinose control) expression vectors (Figure S7, Supporting Information). These were transformed into SHuffle T7 Express competent cells and expressed by autoinduction^[^ ^34^ ^]^ in Luria‐Bertani (LB) medium (10 g L^‐1^ NaCl, 10 g L^‐1^ tryptone, 5 g L^‐1^ yeast extract) 400 mL plus 8 mL 50× auto induction solution (for pET3a 100 mL contains 25  mL glycerol, 2.5 g D‐glucose and 10 g α‐lactose; for TBAD 100 mL contains 25  mL glycerol, 5.0 g D‐glucose plus 5.0 g L‐arabinose) at 30 °C and 200 rpm for 24 h. Cells were pelleted (10 000 × *g*, 4 °C, 10 min) and frozen at ‐20 °C.

Pellets were thawed and resuspended in 20 mL sonication buffer (TBS: 50 × 10^–3^ m Tris, 150 × 10^–3^ m NaCl, pH 8.0; plus: 0.1 × 10^–3^ m ethylenediaminetetraacetic acid (EDTA), 0.5 × 10^‐3^ m phenylmethane sulfonyl fluoride (PMSF), and 1% (v/v) triton x100) and disrupted by sonication on ice (1 s burst, 1 s rest, 15 min). After centrifugation (29 000 × *g*, 4 °C, 10 min) the supernatant was applied to an immobilized metal ion affinity chromatography^[^ ^23^ ^]^ column (Ni‐NTA agarose resin, Qiagen), washed (TBS + 20 × 10^–3^ m imidazole) and the protein eluted (TBS + 300 × 10^–3^ m imidazole) in 2 mL fractions. Pooled fractions were purified by anion exchange on an ÄKTAprime plus (GE Healthcare) by loading onto 5 mL columns from GE Healthcare. A HiTrap DEAE FF low bind anion exchange column was used for small proteins (_pro_HP–DT_prox_ and _pro_HP–DT_dist_) or a HiTrap Q FF high binding column for larger proteins (_pro_GFP–HP–DT_dist_). A gradient between a low salt (50 × 10^–3^ m Tris, 25 × 10^–3^ m NaCl) and high salt buffer (50 × 10^–3^ m Tris, 500 × 10^–3^ m NaCl), with the pH set to ≈1 above the pI of the protein (pH 9.0 for _pro_HP–DT_xxx_, and pH 7.5 for _pro_GFP–HP–DT_dist_), was applied at 2 mL per min for 30 min. Absorbance at 280 nm was monitored as a proxy for protein concentration, and eluted proteins were collected in 2 mL fractions. Fractions were analyzed for purity by sodium dodecyl sulfide polyacrylamide gel electrophoresis (SDS‐PAGE, Figure S8, Supporting Information), buffer exchanged (50 × 10^–3^ m ammonium bicarbonate) and freeze dried. Smaller proteins (_pro_HP–DT_xxx_) could also be purified by RP‐HPLC; and protein purity, mass and oligomeric state was confirmed by nanospray mass spectrometry as described for peptides above.

### Statistical Analysis

Particle sizes were determined from the computationally modeled patches by extrapolating the curvature of the patch and assuming this would form a closed sphere. An average size was calculated by measuring the extrapolated diameter at 19 points equally spaced in time between 5 and 50 ns, and ± one standard deviation of these diameters is the quoted error. The mass of the purified peptide or protein was calculated from the recorded nanospray spectra by fitting using the Micromass MassLynx software maximum entropy analysis MaxEnt1 to 1 Da accuracy. AFM height data were flattened using the first order flattening tool in the Nanoscope software (v1.5), and particle height and diameters extracted using a script available here https://github.com/wells‐wood‐research/galloway‐jg‐hairpin‐self‐assembly‐2020. This script extracted the maximum height (above a 5 nm cutoff) and diameter (above a 50 nm cutoff) of each particle in an AFM topography plot.

## Conflict of Interest

The authors declare no conflict of interest.

## Supporting information

Supporting Information

Supplemental Movie 1

Supplemental Movie 2

Supplemental Movie 3

Supplemental Movie 4

Supplemental Movie 5

## Data Availability

The data that support the findings of this study are openly available from the Leeds University Data Repository at https://doi.org/10.5518/955, reference ^[^ ^35^ ^]^.
